# A one-pot route for the synthesis of Au@Pd/PMo_12_/rGO as a dual functional electrocatalyst for ethanol electro-oxidation and hydrogen evolution reaction†

**DOI:** 10.1039/c9ra06915a

**Published:** 2019-11-18

**Authors:** Ali Ahmadpour, Sara Khadempir, Narges Ashraf, Scott G. Mitchell, Mahdi H. Ahangari

**Affiliations:** Department of Chemical Engineering, Ferdowsi University of Mashhad Mashhad Iran ahmadpour@um.ac.ir; Department of Chemical Engineering, Quchan University of Technology Quchan Iran s.khadempir@qiet.ac.ir; Department of Chemistry, Faculty of Sciences, Ferdowsi University of Mashhad Mashhad Iran; Instituto de Ciencia de Materiales de Aragón (ICMA-CSIC), CISC-Universidad de Zaragoza & CIBER-BBN 50009-Zaragoza Spain

## Abstract

An *in situ* one-pot synthetic route for the synthesis of a Au@Pd/PMo_12_/reduced graphene oxide (rGO) nanocomposite is presented, where the Keggin-type polyoxometalate phosphomolybdic acid (PMo_12_) is used as both reducing and stabilizing agent. High-angle annular dark-field scanning transmission electron microscopy (HAADT-STEM), transmission electron microscopy (TEM), and X-ray diffraction analysis were applied to fully characterize the core–shell structure of Au@Pd/PMo_12_ on the rGO matrix. Electrochemical studies showed how this nanocomposite acts as a dual electrocatalyst for the ethanol electro-oxidation reaction (EOR) and the hydrogen evolution reaction (HER). For the EOR, the Au@Pd/PMo_12_/rGO electrocatalyst offers a low onset potential of −0.77 V *vs.* Ag/AgCl and a high peak current density of 41 mA cm^−2^ in alkaline medium. This feature is discussed *via* detailed cyclic voltammetry (CV) studies illustrating how the superior performance of the synthetic nanocomposite could be attributed to the synergistic effect of Au, Pd, PMo_12_ and rGO. Moreover, it has been confirmed that the proposed electrocatalyst exhibits low overpotentials for 10 mA cm^−2^ current density (*η*_10_) in different pH media. The values of *η*_10_ were −109, 300 and 250 mV *vs.* RHE in acidic, basic and neutral media, respectively. Also, the ability of the electrocatalyst to provide high HER current density and its remarkable stability have been confirmed.

## Abstract 2

Au@Pd/PMo_12_/rGO nanocomposite was synthesized and used as a dual-functional electrocatalyst for HER and EOR.

## Graphical abstract

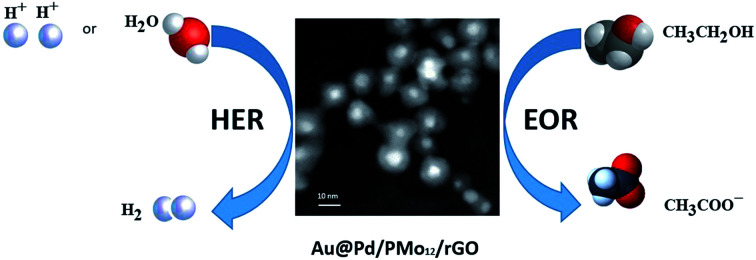

## Introduction

The demand for renewable and green energy resources has risen dramatically in recent years as the negative environmental impact of burning fossil fuels becomes evident. In this regard, small alcohols such as ethanol are currently regarded as some of the most eco-friendly and sustainable energy resources. For ethanol to be used as a fuel, it must be electro-oxidized in direct alcohol fuel cells (DAFCs) that are promising alternative power sources for future portable energy sources and vehicles because of their high energy conversion ability, low emission of pollutants, and low operating temperature. Nevertheless, making them available on the market depends on the innovation of advanced electrocatalyst with excellent activity and durability at an acceptable cost.

On the other hand, hydrogen as a fuel for hydrogen fuel cells is currently viewed as an environment-friendly approach, particularly if derived as a product of a low-cost water splitting cell. Electrochemical hydrogen- and oxygen-evolution reactions are two combined half electrocatalytic reactions that take place in water splitting cells.^1–3^ Nearly all oxygen evolution reaction catalysts are designed for operation in neutral or alkaline media; whereas the most efficient hydrogen evolution reaction catalysts work in acidic media. Consequently, finding applicable catalysts that can simultaneously act over a wide range of pH range and for different reactions is necessary.

Nowadays, dual function (bifunctional) catalysts are routinely developed to promote two distinct electrochemical reactions and mechanisms.^4–6^ Although Pt containing catalysts are of significant interest for the Electrochemical Ethanol Oxidation Reaction (EOR) and Hydrogen Evolution Reaction (HER), its use as a commercial catalyst is limited by its high cost and low earth-abundancy. Pd-based catalytic materials, on the other hand, are viewed as alternative candidates due to their higher earth abundance, good catalytic activity, lower cost, and low poisoning compared with Pt.

Recent studies have demonstrated that incorporating a second metal into Pd nanostructures, giving rise to bimetallic nanoparticles (NPs) such as AuPd, PdNi, PdAg, can significantly improve catalytic activity due to a synergetic effect between two combined metals.^7–12^ Among these bimetallic nanoparticles, AuPd NPs are of special interest because of their high catalytic activity.^13–15^ Currently, there are a number of synthetic procedures towards Pd-based bimetallic NPs such as co-reduction, seed-mediated-growth in aqueous solution, electrodeposition, thermal decomposition, and galvanic replacement reaction.^13–18^ In addition to careful selection of NPs, immobilization on a conductive support improves their effective catalytic performance. Reduced graphene oxide (rGO) nanosheets are two-dimensional carbon materials which offer excellent support for dispersion of NPs, due to their large surface area, superior electronic mobility and thermal conductivity.^19,20^ It should be noted that embedding of pre-synthesized NPs on carbon substrate often causes agglomeration/aggregation therefore finding a direct method synthesis to bimetallic NPs on carbon support is favorable.^15,21^

Herein, we report a simple *in situ* one-pot synthesis of Au@Pd NPs supported on reduced graphene oxide. Precursors of Au and Pd and graphene oxide are simultaneously reduced in the same reaction medium using an anionic molecular metal-oxide – a polyoxometalate (POM) – as a reducing and stabilizing agent. Structural analysis of the nanocomposites reveals an Au-core/Pd-shell structure. The Au@Pd/PMo_12_/rGO nanocomposites display a superior electrocatalytic activity for the HER over a wide range of pH, and also act as an efficient electrocatalyst for EOR in alkaline media. Based on previous results^21^ and the data presented herein, our hypothesis is that the presence of Au promotes the synergetic catalytic activity performance of the Au@Pd/PMo_12_/rGO nanocomposites.

## Experimental section

### Chemicals

Palladium(ii) chloride (PdCl_2_), tetrachloroauric(iii) acid trihydrate (HAuCl_4_·3H_2_O), isopropyl alcohol (C_2_H_6_OH), graphite powder (<50 mm), potassium permanganate (KMnO_4_), potassium persulfate (K_2_S_2_O_8_), phosphorus pentoxide (P_2_O_5_), sodium nitrate (NaNO_3_), hydrogen peroxide (H_2_O_2_), sodium hydroxide (NaOH), ethanol (C_2_H_5_OH), DMF (*N*,*N*-dimethylformamide), sodium dihydrogen phosphate dihydrate (NaH_2_PO_4_·2H_2_O), sulphuric acid (H_2_SO_4_) and commercial Pd/C (1 wt% metal loading) were purchased from Merck (Darmstadt, Germany). All chemicals were in analytical grade and used as received. Phosphomolybdic acid (H_3_PMo_12_O_40_), 10 wt% of commercial Pt/C and Nafion@117 (5 wt% in lower aliphatic alcohols and water) were obtained from Sigma-Aldrich. Al chemicals were in analytical grade and used as received. Phosphate buffer solution (PBS, 0.1 M) was made by dissolving the proper amount of NaH_2_PO_4_·2H_2_O in deionized (DI) water and adjusting the pH to 7 by 0.1 M NaOH (aq) solution.

### Characterization

Scanning electron microscopy (SEM) images were taken using a field emission SEM Inspect F50 with an EDX system INCA PentaFETx3 (FEI Company, Eindhoven, Netherlands) in an energy range between 0–30 keV.

Transmission electron microscopy (TEM) was obtained by a FEI Tecnai T20 microscope operating at 200 kV. High-angle annular dark-field scanning TEM (HAADF-STEM) and STEM mapping were carried out using a Tecnai S-Twin30, 300 keV, GIF-TRIDIEM.

X-ray diffraction (XRD) studies were performed on X-ray diffractometer X'Pert Pro MPD (Cu-K_α_ radiation, 40 kV, 40 mA, *λ* = 1.54060 Å)

The composition of the catalyst was examined with the help of inductively coupled plasma mass spectrometry (ICP-MS) by utilizing Agilent ICP-7900.

A μ-Autolab type III electrochemical workstation with a three-electrode cell was used to all electrochemical measurements.

### Synthesis of nanocomposites

Graphene oxide (GO) was prepared based on modified Hummers' method as illustrated in our previous report.^21^ This process includes two chemical oxidation steps for converting the graphite powder to GO. GO was utilized as a matrix for sketching the Au@Pd/PMo_12_/rGO and Pd/PMo_12_/rGO nanocomposites; that were synthesized *via* the photocatalytic polyoxometalate assisted method. In a typical synthesis, 0.67 mg PdCl_2_, 1.6 mg HAuCl_4_·3H_2_O and 27.4 mg PMo_12_ – as reducing and stabilizing agent-were added to 100 mL DI water and finally dissolved. Then, 10 mL of the as-prepared solution was transferred to a spectrophotometer cell, 480 μg GO (20 wt% loading) was added to the mixture and ultrasonicated for 20 min. Thereafter, 2 mL isopropyl alcohol-as sacrificial agent-were added and the entire reaction mixture was irradiated by a high pressure mercury vapour UV lamp (125 W) for 2 h under magnetic stirring. The Au@Pd/PMo_12_/rGO nanocomposite was obtained after successive washing, centrifuging and drying process. The atomic ratio of Au to Pd was set to (1 : 1) and also, the amount of GO was adjusted based on the different loading of metal nanoparticles on GO (10, 20, 30 and 40 wt%). The Pd/PMo_12_/rGO nanocomposite was produced with the same procedure without addition of HAuCl_4_·3H_2_O salt solution.

### Working electrode preparation

The catalyst ink was prepared by the following procedure: the proper amount of the as-prepared dried nanocomposite was dispersed in DI water to achieve the aqueous suspension of the catalyst ink (1.0 mg mL^−1^).

Glassy carbon electrode (GCE, 2 mm in diameter, Azar Electrode Co.) was used as working electrode which was pre-treatment by surface polishing with 0.05 μm alumina slurry, followed by ultrasonication in ethanol and then in water. To fabricate the modified GCE, 3 μL of catalyst ink was drop casted on GCE and then dried at ambient temperature.

### Electrochemical measurements

The HER and EOR were performed by a three-electrode cell with the saturated Ag/AgCl electrode (Azar Electrode Co.) as the reference electrode, platinum wire (Azar Electrode Co.) as the counter electrode and coated glassy carbon electrode (GCE) was applied as working electrode. The EOR activity of the modified GCE electrodes with the as-prepared electrocatalysts was studied by making cyclic voltammogram (CV) measurements at a scan rate of 50 mV s^−1^ in a mixed solution of 1 M ethanol and 1 M NaOH (aq) as the electrolyte at room temperature. Electrochemical impedance spectroscopy (EIS) was recorded using Ivium Stat (US) with an AC voltage amplitude of 50 mV by scanning frequency from 100 kHz to 10 Hz. The studies of HER were acquired by linear sweep voltammetry (LSV) experiments which was carried out at the scan rate of 50 mV s^−1^ in an electrolyte solution of 0.5 M H_2_SO_4_ (pH 0.3), 1.0 M NaOH (pH 14) and 0.1 M PBS (pH 7). The LSV results were reported with respect to the reversible hydrogen electrode (RHE) potential, which was described by the following equation:*E*_RHE_ = *E*_Ag/AgCl_ + 0.059 × pH + 0.197

## Results and discussion

### Structural analysis

**Fig. 1:**
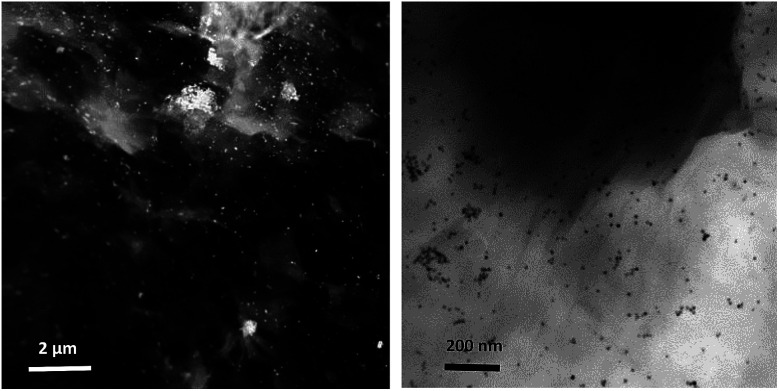
HAADF-STEM (left) and TEM (right) images of Au@Pd/PMo_12_/rGO.

**Fig. 2:**
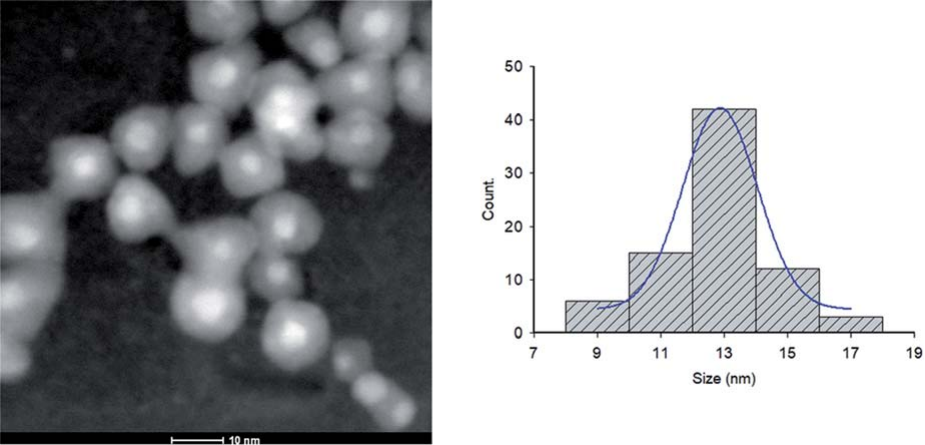
High magnification HAADF-STEM image and the corresponding size distribution histogram of Au@Pd/PMo_12_/rGO nanohybrid.

NPs distribution and surface structure of 20 wt% loading of Au@Pd NPs on rGO were analyzed by HAADF-STEM, TEM and SEM. The corresponding low-magnification HAADF-STEM and TEM images confirm the uniform dispersion of the NPs on GNSs (Fig. 1). Moreover, high-magnification HAADF-STEM (Fig. 2) of the as-prepared nanocomposite shows a clear contrast between the heavier and the lighter elements in the NPs that demonstrate the Au core–Pd shell nanostructure with an average size of 13 nm. The spherical Au cores possess an average diameter of 4 nm and are surrounded by 4 nm thick Pd shells.

**Fig. 3:**
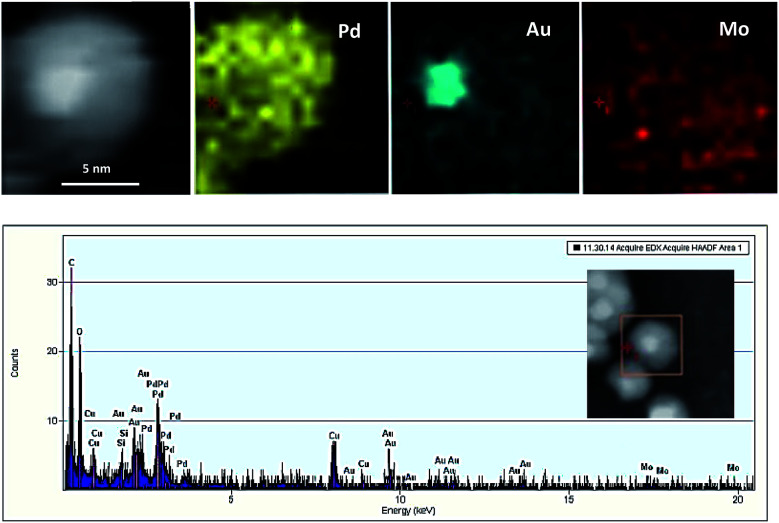
EDS elemental mapping of Au@Pd/PMo_12_/rGO and accompanying EDS spectrum.

EDS elemental analysis maps and EDS spectra (Fig. 3) show Au atoms are mainly located in the center of the particles whereas Pd atoms are comprised a complete shell around the core, whenever a minor Au population are visible in the shell.

The Mo from the PMo_12_ was found entirely covering the crystalline NPs providing evidence for their stabilizing role.

The exact composition of Au@Pd NPs was identified to be Au_41_Pd_59_ (atomic ratio of Au : Pd = 1 : 1 in precursor) by ICP-MS analysis. Also the total metal loading on GO was found (18.7 wt%) which is closed to the initial metal loading (20 wt%).

**Fig. 4:**
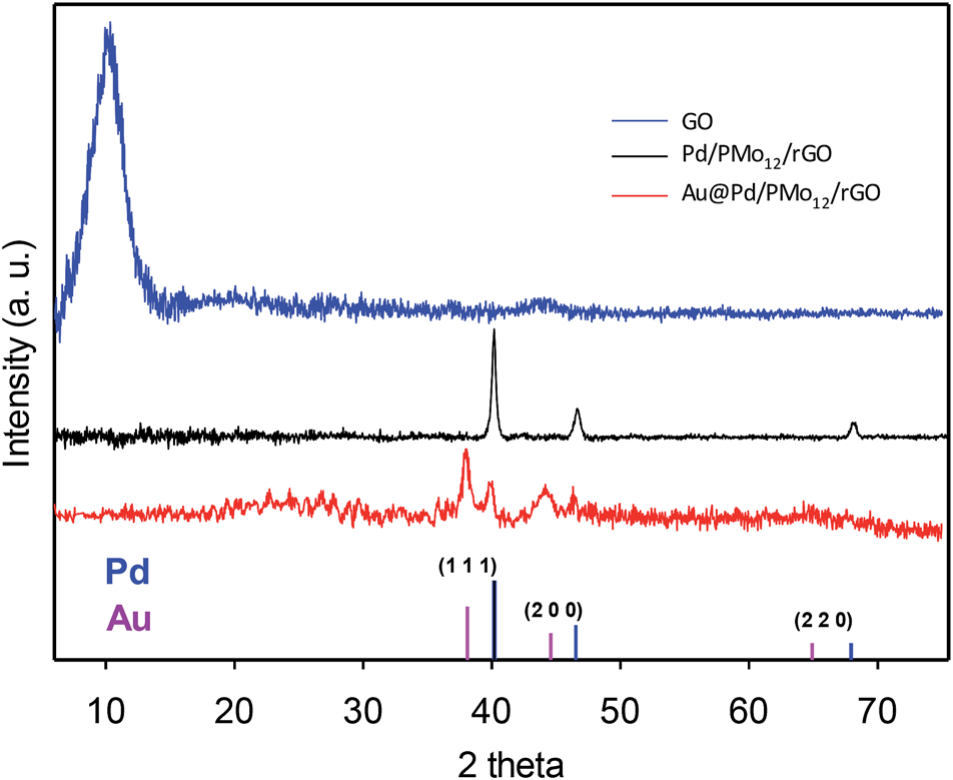
XRD pattern of GO, Pd/PMo_12_/rGO and Au@Pd/PMo_12_/rGO. A standard XRD pattern of Pd and Au is given.

The crystalline nature of the Au@Pd NPs and simultaneous reduction of the as-made GO into rGO can be verified by XRD analysis. Fig. 4 (XRD pattern of the GO) shows the existence of diffraction peak at 10.22° (8.65 °A interlayer *d*-spacing) inferred the successful oxidation *via* the intercalation of the oxygen-containing functional groups in the interlayer of graphite powder and their exfoliation.^22–25^ When the Au@Pd NPs or Pd NPs were loaded onto the graphene surfaces, the sharp peak at 10.22° vanished turning into a broader peak at around 20–30°, meaning that the graphitic nature was reconstructed by the PMo_12_ simultaneous reduction process. In addition, the Au@Pd/PMo_12_/rGO pattern shows well-separated reflection peaks in (1 1 1) and (2 0 0) planes and nearly unclear peaks in (2 2 0) plane attributing to crystalline fcc structure for both of Pd and Au elements, which represents additional confirmation that Au@Pd NPs include a bimetallic core–shell structure and do not involve a homogenous bimetallic alloy.^16,18,26^

Furthermore, the Pd (1 1 1) peak shifted *ca.* 0.3° to lower two theta angle with respect to the Pd (1 1 1) peak position in the Pd/PMo_12_/rGO pattern (40.172° to 39.84°, corresponding to a *d*-spacing change from 2.240 Å to 2.260 Å) which indicates a 0.88% lattice expansion. Such phenomena in bimetallic systems have been referred to the “strain effect” that originates from the substantial lattice mismatch and interaction between two neighboring metals.^18,26–28^ Although an intact Pd shell is assembled, the Au core atoms are able to stretch the Pd atomic shell making the surface electronic structure different from the monometallic and unstrained Pd. This property can be favorable for EOR, as discussed later.^29–31^ Moreover, there are no visible diffraction peaks of the PMo_12_, inferring highly dispersed amorphous PMo_12_ in the nanocomposite structure.

Another experiment was conducted to further check the presence of PMo_12_ in the Au@Pd/PMo_12_/rGO nano-structure. Since PMo_12_ are hydrolyzed in pure aqueous solvent, the cyclic voltammetry behavior of PMo_12_ and Au@Pd/PMo_12_/rGO were investigated in 50% v/v water : DMF solution containing 0.5 M H_2_SO_4_ as an electrolyte.

**Fig. 5:**
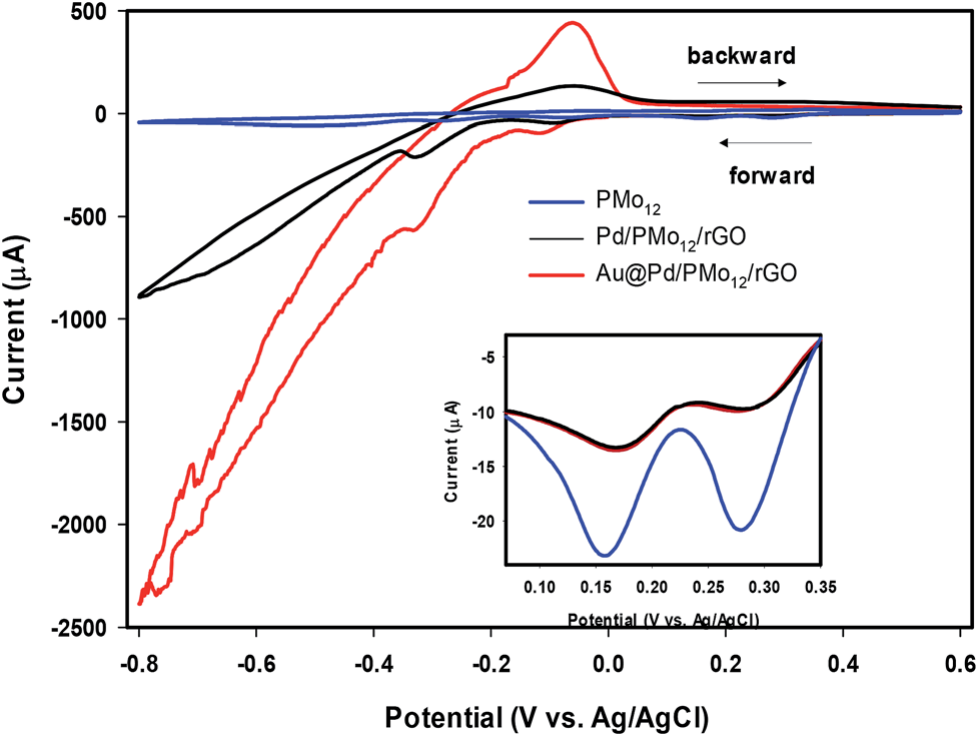
Cyclic voltammograms (CVs) of Au@Pd/PMo_12_/rGO, Pd/PMo_12_/rGO and pure PMo_12_ in 50% (v/v) water–DMF solution containing 0.5 M H_2_SO_4_.

It should be considered that PMo_12_ can undergo a multi-electron reversible redox process without losing its integrity.^32–35^ By comparing the CVs of PMo_12_, Pd/PMo_12_/rGO and Au@Pd/PMo_12_/rGO (Fig. 5), it can be inferred that the cathodic voltammetric peaks in the forward scan at *ca.* 0.31 and 0.19 V in Pd/PMo_12_/rGO and Au@Pd/PMo_12_/rGO electrode should be originated from the characteristic redox behavior of PMo_12_ and verifies that PMo_12_ is adsorbed on Pd during the synthesis of the nanohybrides (inset in Fig. 5). The other characteristic peaks of PMo_12_ overlap by hydrogen evolution region.

### EOR analysis

**Fig. 6:**
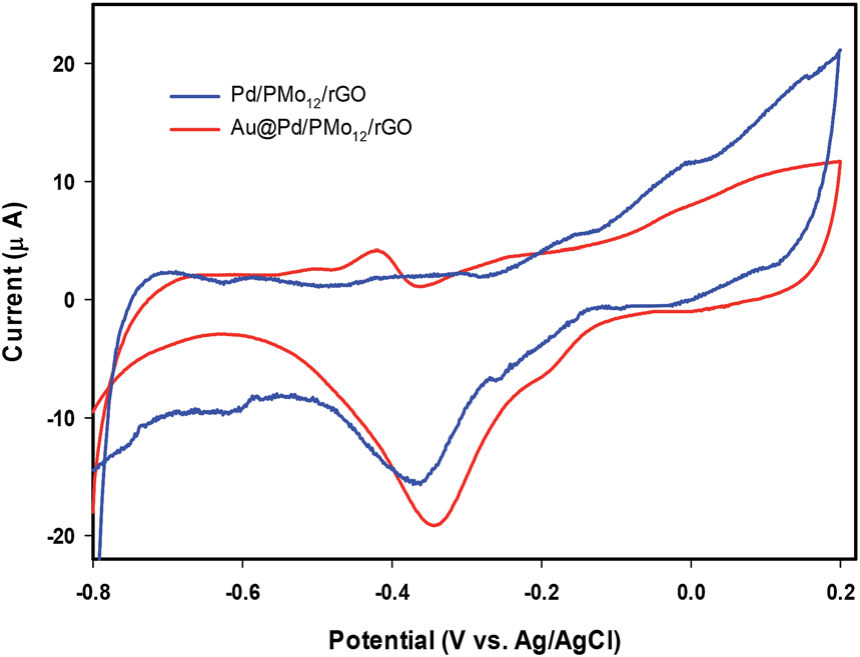
CV of Au@Pd/PMo_12_/rGO and Pd/PMo_12_/rGO in 1 M NaOH solution at a scan rate of 50 mV s^−1^.

**Fig. 7:**
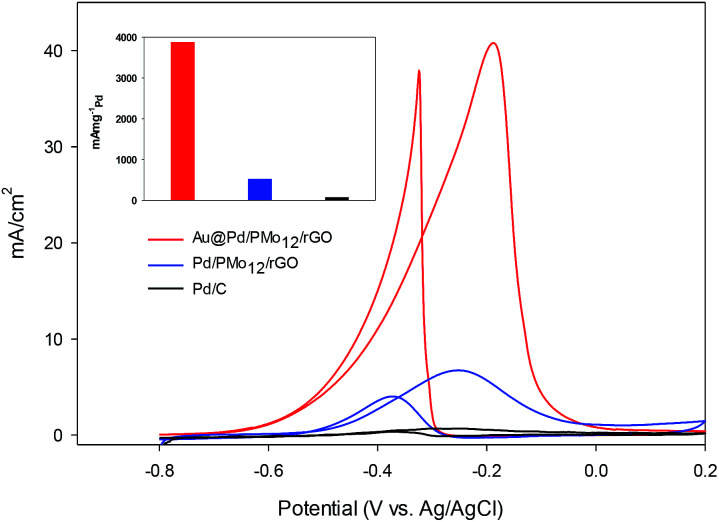
CV curves for Au@Pd/PMo_12_/rGO and Pd/PMo_12_/rGO and Pd/C in 1 M NaOH + 1 M ethanol at a scan rate of 50 mV s^−1^.

In an effort to evaluate the electrocatalytic activity of the as-prepared nanocomposite, a series of CV and CA experiments were carried out in 1 M NaOH and 1 M NaOH + 1 M ethanol solution. The electrochemical behavior of Au@Pd/PMo_12_/rGO in aqueous 1 M NaOH solution as supporting electrolyte is shown in Fig. 6. For comparison, the CV response of Pd/PMo_12_/rGO is also given. Different potential regions can be recognized for the Au@Pd/PMo_12_/rGO nanocomposite, which indicates different electrochemical processes occurring on the surface of the electrocatalyst.^29^ During the forward scan; the potential region between −0.8 V and −0.35 V *vs.* Ag/AgCl is ascribed to OH^−^ ion adsorption on Pd to form oxygen-containing species (Pd–OH_ads_) which is partially overlapped by the hydrogen desorption process. Another potential area emerged at −0.35 V corresponding to Pd oxidation to its metal oxide on the catalyst surface. Clearly, a single reduction peak at *ca.* −0.3 V in the backward scan associated to the Pd oxide reduction.^14,36–38^ Fig. S1 and S2† show the CV responses towards EOR and TEM images of different Au@Pd NPs loading on GO (10, 20, 30 and 40 wt% loading). Based on these results, EOR was boosted by increasing metal loading till 20 wt% loading and then decreased with further loading *via* the agglomeration process, so more experiments were done on 20 wt% loading of Au@Pd/PMo_12_/rGO. Fig. 7 shows the CV responses of the 20 wt% loading of Au@Pd/PMo_12_/rGO during EOR. Also, the CV responses of Pd/PMo_12_/rGO as well as commercial Pd/C are included for the purpose of comparison. It is notable that based on our previous results, Pd/PMo_12_/rGO exhibited remarkable catalytic activity toward EOR in 1 M NaOH.

The strong oxidation peaks in the positive sweep with *E*_p_ at *ca.* −0.19 V for Au@Pd/PMo_12_/rGO and *ca.* −0.25 V for Pd/PMo_12_/rGO represent the ability of both Pd-based electrocatalysts towards EOR. As can be seen, the current density of Au@Pd/PMo_12_/rGO is considerably higher than that seen for Pd/PMo_12_/rGO (*i*_p_ ∼ 41 *vs.* 7 mA cm^−2^). The onset potential for Au@Pd/PMo_12_/rGO is observed at *ca.* −0.77 V that shifts to more positive potentials (*ca.* −0.65 V) for Pd/PMo_12_/rGO. In the reverse scan, another oxidation process also occurred that corresponds to the irreversible EOR. It should be noted that the onset potential of the reverse peak is near to the onset potential for the reduction of Pd oxide in 1 M NaOH supporting electrolyte mentioned in Fig. 6.

**Fig. 8:**
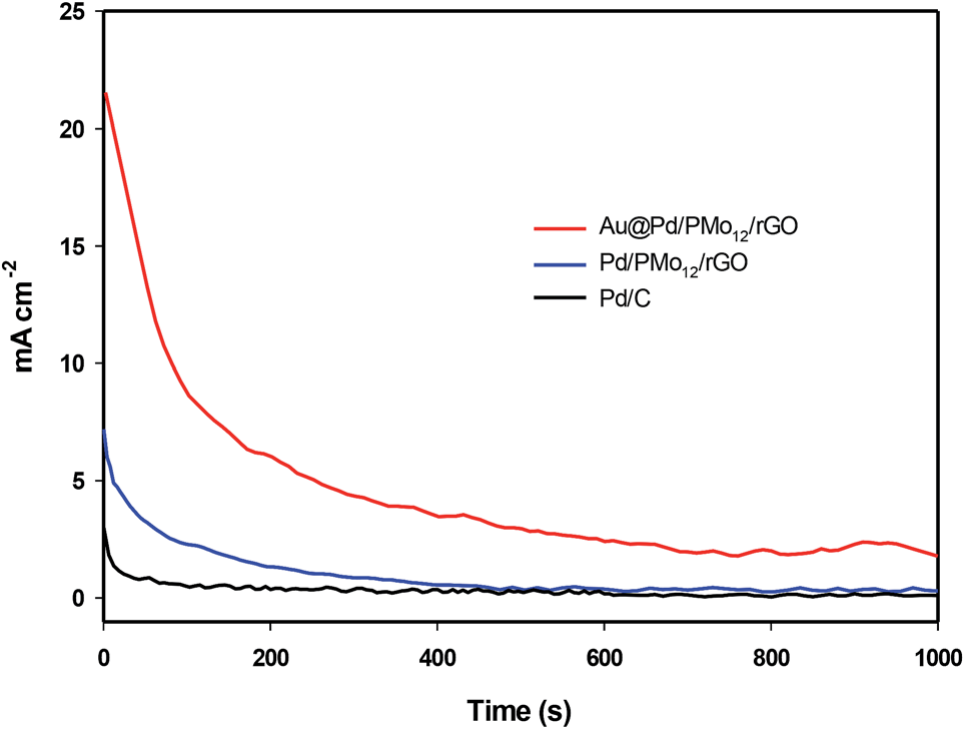
Chronoamperograms of Au@Pd/PMo_12_/rGO and Pd/PMo_12_/rGO and Pd/C in at −0.3 V in 1 M NaOH + 1 M ethanol solution.

The electrochemical stability of the as-prepared nanocomposites is examined by chronoamperometry measurement (*j*–*t*) at a fixed potential of −0.3 V *versus* Ag/AgCl in 1 M NaOH + 1 M ethanol solution (Fig. 8). It is clear that the current drops is slower on the Au@Pd/PMo_12_/rGO than on the Pd/PMo_12_/rGO and Pd/C electrodes which was associated to the poisoning of the electrode by intermediate species. The result show that core@shell structure of Au and Pd can improve not only the superior electrocatalytic activity but also the electrocatalytic stability toward EOR. The electrochemical surface area (ECSA) was calculated based on the columbic charge collected during the reduction region of Pd oxide to Pd (see Fig. 6) by considering that the charge consumption for monolayer of Pd oxide reduction is 405 μC cm^−2^; and then corrected to the mass of Pd that used for electrode fabrication. Eqn (1) shows the ECSA formula:^39^1
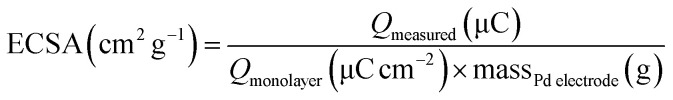


The estimated ECSA for the Au@Pd/PMo_12_/rGO and Pd/PMo_12_/rGO were 56 and 38 m^2^ g_Pd_^−1^, respectively. As a consequence, the 1.45-fold increase in ECSA is mostly attributed to bimetallic Au@Pd NPs, because the distribution and carrier effect of graphene are the same in each of the two electrocatalysts.

The remaining section is focused on the probable reasons for the remarkable catalytic activity of the Au@Pd/PMo_12_/rGO nanocomposite. For this purpose, the EOR mechanism that discussed in the literatures is presented in the following equations.^37,38^

2 Pd + CH_3_CH_2_OH ↔ Pd–(CH_3_CH_2_OH)_ads_

3 Pd–(CH_3_CH_2_OH)_ads_ + 3OH^−^ → Pd–(CH_3_CO)_ads_ + 3H_2_O + 3*e*^−^

4 Pd–(CH_3_CO)_ads_ + Pd–OH_ads_ → Pd–CH_3_COOH + Pd

5 Pd–CH_3_COOH + OH^−^ → Pd + CH_3_COO^−^ + H_2_O

It is revealed that the ethanol was adsorbed in the initial potential region and turned into the resultant ethoxy groups at the surface of Pd (eqn (2) and (3)). Eqn (4) points out the stripping off the Pd surfaces by Pd–OH_ads_ and eqn (5) involves the fast releasing of the acetate as the major product. It is worth noting that among the entire process, eqn (4) is the rate-determining step meaning that any strategies that facilitate the formation and effective coverage of CH_3_CO_ads_ and OH_ads_ species on the surface of catalyst can promote the EOR.^35,37,38^

Based on this mechanism, one reason for improved catalytic activity of the Au@Pd/PMo_12_/rGO could be due to the geometric *“strain effect”* observed from the XRD data. It is entirely possible that the Au core atoms can modify the surface electronic structure of Pd and thereby can enhance the adsorption of OH_ads_ on Pd surface and accelerate the dissociative adsorption of ethanol on Au@Pd/PMo_12_/rGO electrocatalyst *via*eqn (5). Moreover, the ligand effect – the heterometallic bonding interaction between Pd and Au – may further influence the superior catalytic activity of the Au@Pd/PMo_12_/rGO.^29,40–42^ All of the above reasons can justify the negative shift in the onset potential EOR and also higher current density of Au@Pd/PMo_12_/rGO compared to the unstrained Pd in Pd/PMo_12_/rGO electrocatalyst.

**Fig. 9:**
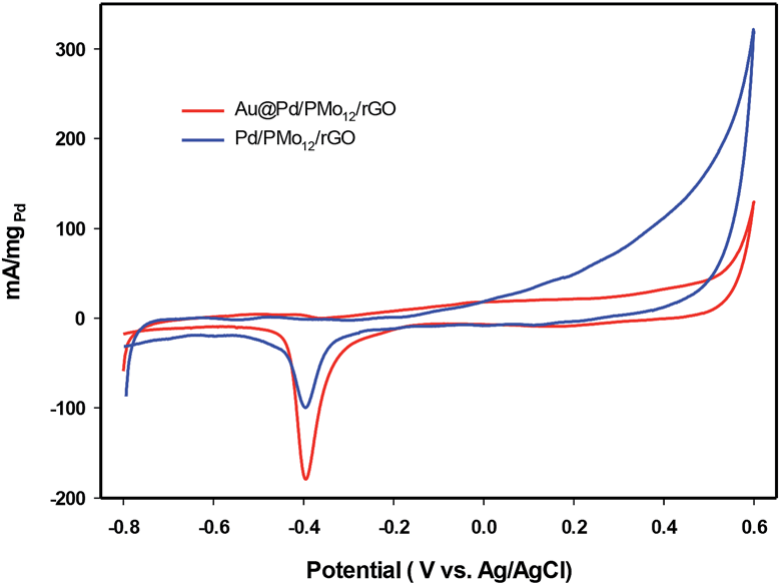
CV curves for Au@Pd/PMo_12_/rGO and Pd/PMo_12_/rGO in 1 M NaOH at a scan rate of 50 mV s^−1^.

Another reason for better catalytic activity of the nanocomposite could be related to lower poisoning effect. Based on the EOR mechanism, poisoning effect which originates from Pd oxide formation, can block the active sites of the electrocatalyst. The presence of Au confers a delay in Pd oxide formation. Fig. 9, indicates the CVs of the Au@Pd/PMo_12_/rGO and Pd/PMo_12_/rGO in 1 M NaOH electrolyte. It shows that the Pd oxide formation is slowed significantly in the Au@Pd/PMo_12_/rGO compared to the Pd/PMo_12_/rGO electrocatalyst. The results confirm that fortunately the poisoning effect of the oxide formation can be manipulated by incorporation of the second metal and the Au@Pd/PMo_12_/rGO structure can inhibit the Pd oxide formation which leads to improved EOR.

**Fig. 10:**
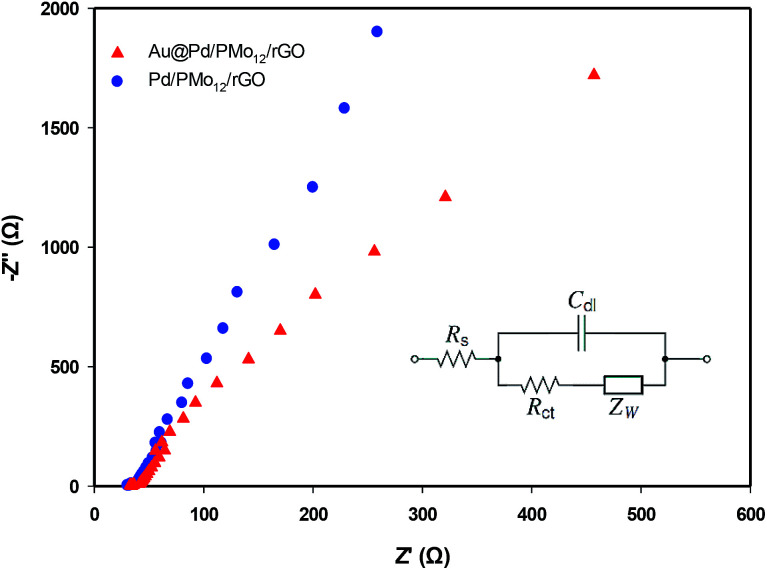
Nyquist plots of EOR on the Au@Pd/PMo_12_/rGO and Pd/PMo_12_/rGO electrodes in 1 M NaOH + 1 M ethanol solution at −0.17 V *vs.* Ag/AgCl; inset is an equivalent Randles circuit.

In order to probe the electrochemical charge transfer properties of the electrocatalyst, the EIS technique was applied. In general, the Nyquist plot is related to the charge transfer resistance (*R*_ct_) of the electrochemical reaction in high frequency region and Warburg impedance in low frequency zone.^43–45^Fig. 10 represents the Nyquist plots of the Au@Pd/PMo_12_/rGO and Pd/PMo_12_/rGO to evaluate the kinetics of electrocatalyst in EOR. In both plots, the semicircular zone is not well defined leading to a dominance of the Warburg impedance over the whole available range. So, the system is kinetically facile and mass transfer plays a role in EOR.

The Randles equivalent circuit (inset in Fig. 10) was fitted based on the EIS data, which contains the elements of: ohmic solution resistance (*R*_s_), Warburg impedance (*Z*_W_), (*R*_ct_) and double layer capacitance (*C*_dl_).^46,47^ According to this equivalent circuit, the calculated *R*_ct_ for Au@Pd/PMo_12_/rGO and Pd/PMo_12_/rGO electrodes are 773 Ω cm^2^ and 1270 Ω cm^2^, respectively. It is noted that the same *R*_s_ = 33 Ω was calculated. The higher charge transfer rate demonstrating that the EOR was more kinetically facile on the Au@Pd/PMo_12_/rGO electrode than on the Pd/PMo_12_/rGO which is attributed to the interaction between two neighbouring metals in Au@Pd nanostructure.

It should be noted that the electrocatalytic experiments were performed in alkaline media and PMo_12_ may decompose at this pH;^34,48,49^ however, no significant effect on the catalytic activity of the Au@Pd/PMo_12_/rGO was observed, even following several runs.

### HER study

The HER performance of electrocatalysts commonly examined by LSV method which is displayed as the current density (normalized to the surface area of the GCE) *versus* applied potential.

The overall HER in acidic medium is2H_3_O^+^ + 2*e*^−^ → H_2_ + 2H_2_Oand in neutral or basic solution is2H_2_O + 2*e*^−^ → H_2_ + 2OH^−^.

**Fig. 11:**
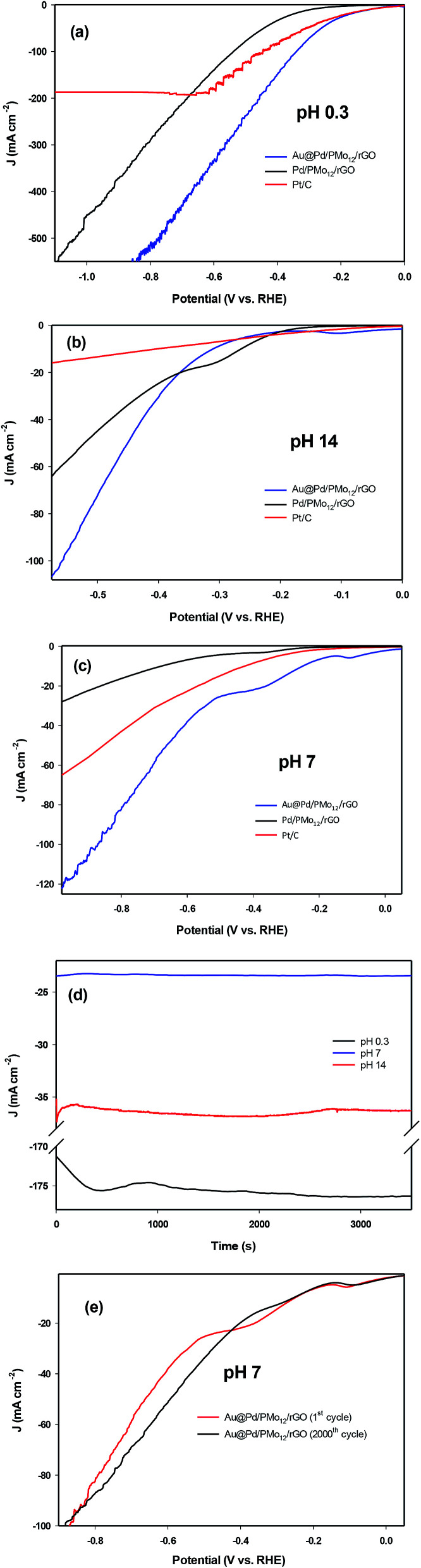
LSV curves (scan rate 50 mV s^−1^) in (a) 0.5 M H_2_SO_4_ (pH 0.3), (b) 1 M NaOH (pH 14), (c) 0.1 M PBS (pH 7), (d) chronoamperograms of Au@Pd/PMo_12_/rGO at −0.4 V (RHE) in different pH media, (e) 0.1 M PBS (pH 7) on Au@Pd/PMo_12_/rGO in first cycle and after 2000 cycles.

Although the kinetics of HER in neutral medium are slower than in acidic or basic media in principle, neutral HER is of interest in commercial scale hydrogen production because of the gentle reaction condition and stability against corrosion, oxidation *etc*^1,50,51^. The LSV curves for the as-prepared Au@Pd/PMo_12_/rGO, Pd/PMo_12_/rGO and PMo_12_/rGO nanocomposites at different pH values are shown in Fig. 11a–e. For comparison purposes, the HER activity of commercial Pt/C were also evaluated. The reason why Pt/C was selected instead of pure Pt electrode is that the Pt has a similar catalytic activity with Pt/C in acidic medium, but it has a much lower activity than Pt/C in neutral and basic media which is because Pt is a bulk catalyst, whereas Pt/C contains Pt nanoparticles as catalyst. Fig. 11a shows the LSV curves under acidic condition (0.5 M H_2_SO_4_, pH 0.3). Here, the required overpotential for 10 mA cm^−2^ current density (*η*_10_) is considered as the criterion for comparing HER electrocatalytic activity. The Au@Pd/PMo_12_/rGO shows much lower *η*_10_ (−109 mV *vs.* RHE) than that of Pd/PMo_12_/rGO (−295 mV *vs.* RHE) and near to that of Pt/C (−80 mV *vs.* RHE). Furthermore, it is very interesting that the proposed electrocatalyst is able to carry large current densities of more than 500 mA cm^−2^ for HER. These evidences manifest that the HER kinetics on Au@Pd/PMo_12_/rGO is very fast compared to Pd/PMo_12_/rGO, showing its excellent electrocatalytic activity.

Fig. 11b represents the LSV curves in basic medium (1 M NaOH). The Au@Pd/PMo_12_/rGO shows a boosted electrocatalytic activity; as the *η*_10_ of Pd/PMo_12_/rGO is 280 mV (*vs.* RHE) which is lower than that of Au@Pd/PMo_12_/rGO (300 mV *vs.* RHE) and Pt/C (401 mV *vs.* RHE). But, the Au@Pd/PMo_12_/rGO delivers greatly higher current density (more than 120 mA cm^−2^) compared to Pd/PMo_12_/rGO and Pt/C which means the greater catalytic performance of Au@Pd/PMo_12_/rGO.

Fig. 11c shows the LSVs curves in neutral solution (PBS, 0.1 M, surprisingly, the Au@Pd/PMo_12_/rGO electrocatalyst needs only 250 mV (*vs.* RHE) overpotential to drive 10 mA cm^−2^ whereas the Pt/C and Pd/PMo_12_/rGO takeover 430 and 677 mV (*vs.* RHE), respectively. Fig. 11d presents the stability of HER on the Au@Pd/PMo_12_/rGO electrocatalyst under a fixed potential of −0.4 V (*vs.* RHE) at pH = 0.3, 7 and 14. The time depended current density curves show that the current densities were almost unchanged at pH 7 and 14 during the 3600 s chronoamperometric test. Furthermore, at pH 0.3, the current density was increased within the first 2500 s and then it remains nearly constant.

Since the activity of electrocatalyst was very important in neutral solution, the durability of the electrocatalyst was explored by using continuous LSV scans in 0.1 M PBS. As represented in Fig. 11e the Au@Pd/PMo_12_/rGO electrocatalyst maintains a comparable performance to the first cycle after 2000 cycles, such that there is only 1.6% increase in *η*_10_; suggesting an excellent durability in neutral solution. All of the above evidences revealed that the Au@Pd/PMo_12_/rGO is an efficient electrocatalyst for HER.

## Conclusions

To sum up, Au@Pd NP-decorated rGO was synthesized using PMo_12_ as both reducing and stabilizing agent. The core@shell structure of the Au@Pd has been confirmed by TEM, XRD, HAADF-STEM imaging and EDS elemental mapping. The superior electrocatalytic behavior of the Au@Pd/PMo_12_/rGo compared to Pd/PMo_12_/rGo towards EOR was proved by electrochemical methods. The enhanced performance could be attributed to the incorporation of Au core resulting from: (1) geometric “*strain effect*” (lattice mismatch) and ligand effect that facilitate OH^−^ adsorption on Pd (2) limiting the PdO formation (3) higher ECSA. Also, the HER electrocatalytic performances of Au@Pd/PMo_12_/rGo in acidic, alkaline and neutral media were demonstrated. The proposed electrocatalyst exhibits remarkably high activity when compared to the commercial Pt/C and Pd/PMo_12_/rGo.

## Conflicts of interest

There are no conflicts to declare.

## Supplementary Material

RA-009-C9RA06915A-s001
