# Supplementary material for: A one-pot route for the synthesis of Au@Pd/PMo12/rGO as a dual functional electrocatalyst for ethanol electro-oxidation and hydrogen evolution reaction
Source: RSC Adv. 2019 Nov 18;9(64):37537–45. doi: 10.1039/c9ra06915a (PMC9075539; doi:10.1039/c9ra06915a)
Supplement: RA-009-C9RA06915A-s001 [file RA-009-C9RA06915A-s001.pdf]

Electronic Supplementary Material (ESI) for RSC Advances.
This journal is © The Royal Society of Chemistry 2019

Electronic Supplementary Information

A One-Pot Route to the Synthesis of Au@Pd/PMo_{12}/rGO as a Dual Functional Electrocatalyst for Ethanol Electro-oxidation and Hydrogen Evolution Reaction

Ali Ahmadpour [a,*], Sara Khadempir [b,*], Narges Ashraf [c], Mahdi H. Ahangari [a], Scott G. Mitchell [d]

[a] Department of Chemical Engineering, Ferdowsi University of Mashhad, Mashhad, Iran.

[b] Department of Chemical Engineering, Quchan University of Technology, Quchan, Iran.

[c] Department of Chemistry, Faculty of Sciences, Ferdowsi University of Mashhad, Mashhad, Iran.

[d] Instituto de Ciencia de Materiales de Aragón (ICMA-CSIC), CISC-Universidad de Zaragoza & CIBER-BBN, 50009-Zaragoza, Spain.

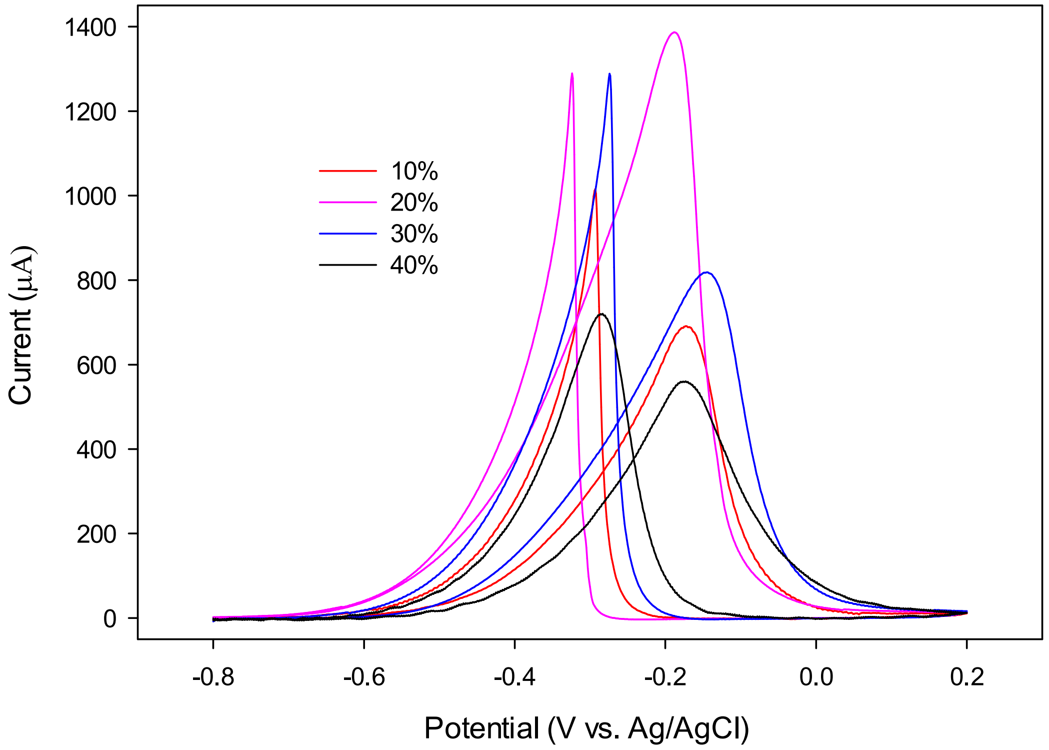

Fig.S1: Cyclic voltammetric curves for Au@Pd/PMo_{12}/rGO at different loading of Au@Pd NPs on rGO in 1.0 M NaOH + 1.0 M ethanol, scan rate: 50 mV s^{-1}

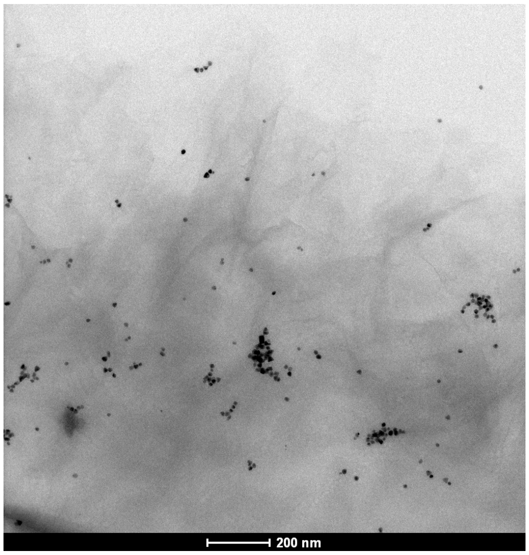

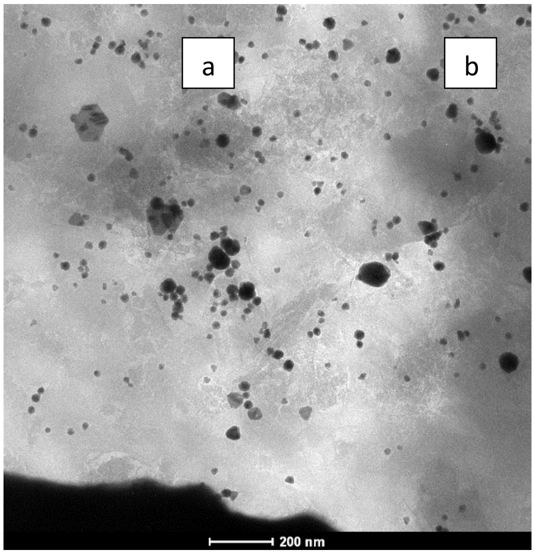

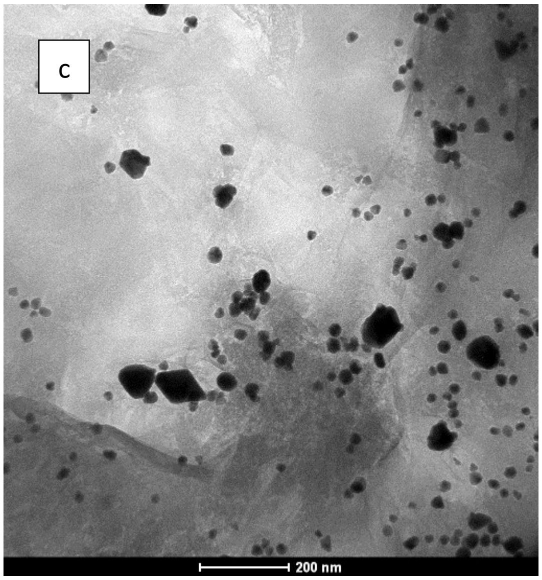

Fig. S2: TEM images of Au@Pd/PMo_{12}/rGO nanohybrid with different loading of Au@Pd NPs rGO, (a) 10%, (b) 30% and (c) 40%.
